# Effects of physical activity on cognitive function in healthy older women: a systematic review and meta-analysis

**DOI:** 10.3389/fnagi.2026.1928056

**Published:** 2026-10-15

**Authors:** Yong Fan, Jiqiao He, Zhicheng Zhu, Youjia Mao, Lisha Xie, Cui Huang, Zijian Zhu

**Affiliations:** 1 Institute of Physical Education, Xinyu University, Xinyu, China; 2 School of Sports Medicine and Health, Chengdu Sport University, Chengdu, China

**Keywords:** brain-derived neurotrophic factor, cognitive function, Mini-Mental State Examination, older women, physical activity

## Abstract

**Background:**

Age-related cognitive decline represents a major public health challenge, particularly among older women who face a disproportionately elevated risk of dementia. Physical activity has been proposed as a modifiable lifestyle factor with potential neuroprotective benefits; however, evidence specific to cognitively healthy older women remains limited.

**Methods:**

A systematic review and meta-analysis of randomized controlled trials (RCTs) was conducted following the Preferred Reporting Items for Systematic Reviews and Meta-Analyses (PRISMA) guidelines. PubMed, the Cochrane Library, Web of Science, and Embase were searched from database inception to 26 September 2026. Studies were eligible if they enrolled healthy women aged >60 years and compared physical activity interventions with control conditions. The primary outcome was global cognitive function assessed using the Mini-Mental State Examination (MMSE) or the Montreal Cognitive Assessment (MoCA). Secondary outcomes included brain-derived neurotrophic factor (BDNF) and body mass index (BMI). Pooled effect sizes were calculated using random-effects or fixed-effect models as appropriate. Subgroup analyses and meta-regression were performed to explore potential moderators. The certainty of evidence was appraised using the Grading of Recommendations, Assessment, Development and Evaluations (GRADE) approach.

**Results:**

Ten RCTs encompassing 446 participants were included. Physical activity was associated with higher global cognitive function scores (Hedges’ g = 0.63, 95% CI: 0.12–1.13, *p* = 0.02) and increased BDNF levels (MD = 2.43, 95% CI: 0.57–4.28, *p* = 0.01). No statistically significant effect on BMI was observed (MD = 0.63, 95% CI: −1.12–2.37, *p* = 0.48). Substantial between-study heterogeneity was observed for global cognitive function (I^2^ = 77.82%). Formal tests for subgroup differences were not statistically significant for either intervention duration (*p* = 0.82) or intervention frequency (*p* = 0.12).

**Conclusion:**

The pooled evidence suggests potential beneficial effects of physical activity on global cognitive function and BDNF levels in healthy older women; however, the certainty of evidence was very low. No statistically significant effect on BMI was observed, and subgroup analyses did not provide sufficient evidence that intervention frequency significantly modified the effect on cognitive function. Further well-designed RCTs with larger samples and standardized outcome measures are needed to confirm these findings.

**Systematic review registration:**

PROSPERO under registration number CRD420251249052, https://www.crd.york.ac.uk/PROSPERO/view/CRD420251249052.

## 1 Introduction

With the accelerating global population aging, cognitive decline has emerged as a major public health challenge. Normal aging is accompanied by a range of cognitive changes, including reduced processing speed, impaired working memory, and declines in executive function. Although these alterations are distinct from neurodegenerative diseases, inadequate intervention may substantially increase the risk of progression to dementia (Lee and Kim, 2022). Dementia is a syndrome characterized by progressive deterioration in cognitive function, which severely compromises individuals’ ability to perform activities of daily living and imposes a substantial burden on families, healthcare systems, and society as a whole. According to projections from the 2022 Global Burden of Disease Study published in The Lancet Public Health, the number of individuals living with dementia worldwide is expected to increase from 57 million in 2019 to 152.8 million by 2050 (GBD 2019 Dementia Forecasting Collaborators, 2022).

Alzheimer’s disease (AD) is the most common form of dementia and one of the most prevalent neurodegenerative disorders among older adults, accounting for approximately 90% of dementia cases in the older population (Yesavage et al., 1993). AD is characterized by progressive cognitive neurodegeneration accompanied by the loss of synapses and pyramidal neurons (Babic, 1999). Neuropathological changes are primarily concentrated in higher-order functional brain regions, particularly the hippocampus and neocortex, where the extent of damage is most pronounced (De la Rosa et al., 2020). Clinically, AD is characterized by irreversible and progressive deterioration in cognitive, functional, and behavioral capacities (Yesavage et al., 1993). In addition, epidemiological evidence indicates that dementia prevalence is higher in women than in men. Among individuals aged 60–69 years, the prevalence of AD in women has been reported to be approximately 1.9 times that observed in men (Cao et al., 2020). This disparity is largely attributed to women’s longer life expectancy, which increases their susceptibility to age-related diseases and syndromes, including dementia (Plassman et al., 2007).

Given the close association between cognitive decline and dementia, these conditions share numerous risk and protective factors. Some factors are modifiable, including educational attainment, air pollution exposure, smoking, and lifestyle-related behaviors, particularly physical activity (Livingston et al., 2024). In contrast, other factors, such as age, sex, and genetic predisposition—especially the apolipoprotein E ε4 (APOE ε4) allele—are non-modifiable (Molinuevo et al., 2010; Aarsland et al., 2010). From a preventive perspective, greater emphasis should therefore be placed on modifiable risk factors and intervention strategies (Blondell et al., 2014).

Accumulating evidence suggests that physical activity, as a modifiable risk factor and positive lifestyle intervention, may not only reduce the risk of dementia and related neurodegenerative diseases but also help preserve cognitive function during aging (Prakash et al., 2015; Erickson et al., 2013; Scarmeas and Stern, 2003). Numerous studies have demonstrated the beneficial effects of physical activity on preventing cognitive decline in older adults. For example, a study by M. Zhang reported that physical activity significantly improved global cognition, executive function, and memory among healthy adults aged over 60 years (Zhang et al., 2023). Similarly, W. J. Bossers found that combined physical and mind–body exercise interventions effectively enhanced overall cognitive function in older adults both with and without cognitive impairment (Bossers et al., 2015).

Despite the promising clinical potential of physical activity for mitigating age-related cognitive decline, existing research has primarily focused on older adults with dementia, whereas relatively limited attention has been directed toward cognitively healthy older women, despite their substantially higher risk of dementia compared with men (Cao et al., 2020). Moreover, findings from previous studies remain inconsistent. A meta-analysis including 12 prospective studies demonstrated that both low-to-moderate and high levels of physical activity were associated with a reduced risk of cognitive decline among people without dementia (Sofi et al., 2011). In contrast, a study by D. B. Reuben concluded that there remains insufficient evidence from randomized clinical trials to definitively confirm that any intervention can prevent dementia or cognitive decline (Reuben et al., 2024).

Taken together, whether physical activity can effectively improve global cognitive function and thereby help prevent cognitive decline in cognitively healthy older women remains to be comprehensively established. Therefore, the present study conducted a systematic review and meta-analysis of randomized controlled trials to evaluate the effects of physical activity on global cognitive function in healthy older women. In addition, subgroup analyses were performed to examine the potential moderating effects of intervention frequency and duration. The findings of this study may provide evidence-based support for the development of cognitive health promotion strategies targeting older women.

## 2 Methods

This systematic review and meta-analysis was conducted in strict accordance with the Preferred Reporting Items for Systematic Reviews and Meta-Analyses (PRISMA) guidelines and the Cochrane Handbook for Systematic Reviews of Interventions. The study protocol was prospectively registered in the International Prospective Register of Systematic Reviews (PROSPERO; registration number: CRD420251249052).

### 2.1 Eligibility criteria

The eligibility criteria for this systematic review and meta-analysis were established across four domains: study design, participant characteristics, interventions, and outcome measures.

#### 2.1.1 Study design

Only randomized controlled trials (RCTs) were included. Cohort studies, case–control studies, cross-sectional studies, and non-randomized experimental studies were excluded. Reviews and previous meta-analyses were also excluded.

#### 2.1.2 Participants

Studies involving cognitively healthy older women aged >60 years were eligible for inclusion. Studies were excluded if the original trial reported that participants had systemic diseases, including cardiovascular disease, diabetes mellitus, malignant tumors, severe hepatic or renal dysfunction, or psychiatric or neurological disorders such as Alzheimer’s disease or clinically diagnosed depression. Eligibility regarding health status was determined from the inclusion and exclusion criteria and participant characteristics reported in the original studies; the review authors did not independently verify diagnoses or medical records. When the method used by the original study to ascertain disease status was not explicitly reported, no assumption of medical confirmation was made. Studies including participants aged <60 years or male participants without separately reported data for healthy older women were also excluded.

#### 2.1.3 Interventions

Participants in the intervention group received physical activity interventions, including Brain Gym exercises, aquatic strength training, resistance training, traditional resistance exercise, Pilates training, Taekwondo, combined circuit training, and multicomponent exercise programs. Control groups received usual care, maintained their habitual physical activity, or did not participate in any additional structured physical activity. No included control group received structured cognitive training or exercise interventions. Studies in which the intervention group received computer-based cognitive interventions or the control group participated in structured exercise programs were excluded.

#### 2.1.4 Outcomes

The primary outcome was global cognitive function. Secondary outcomes included brain-derived neurotrophic factor (BDNF) levels and body mass index (BMI).

### 2.2 Information sources

A comprehensive literature search was conducted in PubMed, the Cochrane Library, Web of Science, and Embase from database inception to 26 September 2026, without language restrictions. In addition, the reference lists of relevant reviews, systematic reviews, and meta-analyses were manually screened to identify any potentially eligible studies that may have been missed during the electronic database search. All retrieved records were imported into EndNote 25 for duplicate removal and reference management.

### 2.3 Search strategy

The literature search strategy combined controlled vocabulary terms and free-text keywords. The primary search terms included “Exercise,” “Older Women,” and “Cognitive Function,” along with their relevant synonyms and related terms. The search strategy was appropriately adapted according to the indexing characteristics and retrieval requirements of each database. The complete database-specific search strategies, including the search dates and numbers of records retrieved, are provided in Supplementary Table S1.

### 2.4 Selection process

All retrieved records were independently screened by two reviewers at both the title/abstract and full-text stages. Disagreements at either stage were resolved through discussion, and when consensus could not be reached, a third reviewer adjudicated the decision. Inter-rater agreement was not formally quantified using a kappa statistic. The study selection process was documented using the PRISMA flow diagram (Figure 1).

**Figure 1:**
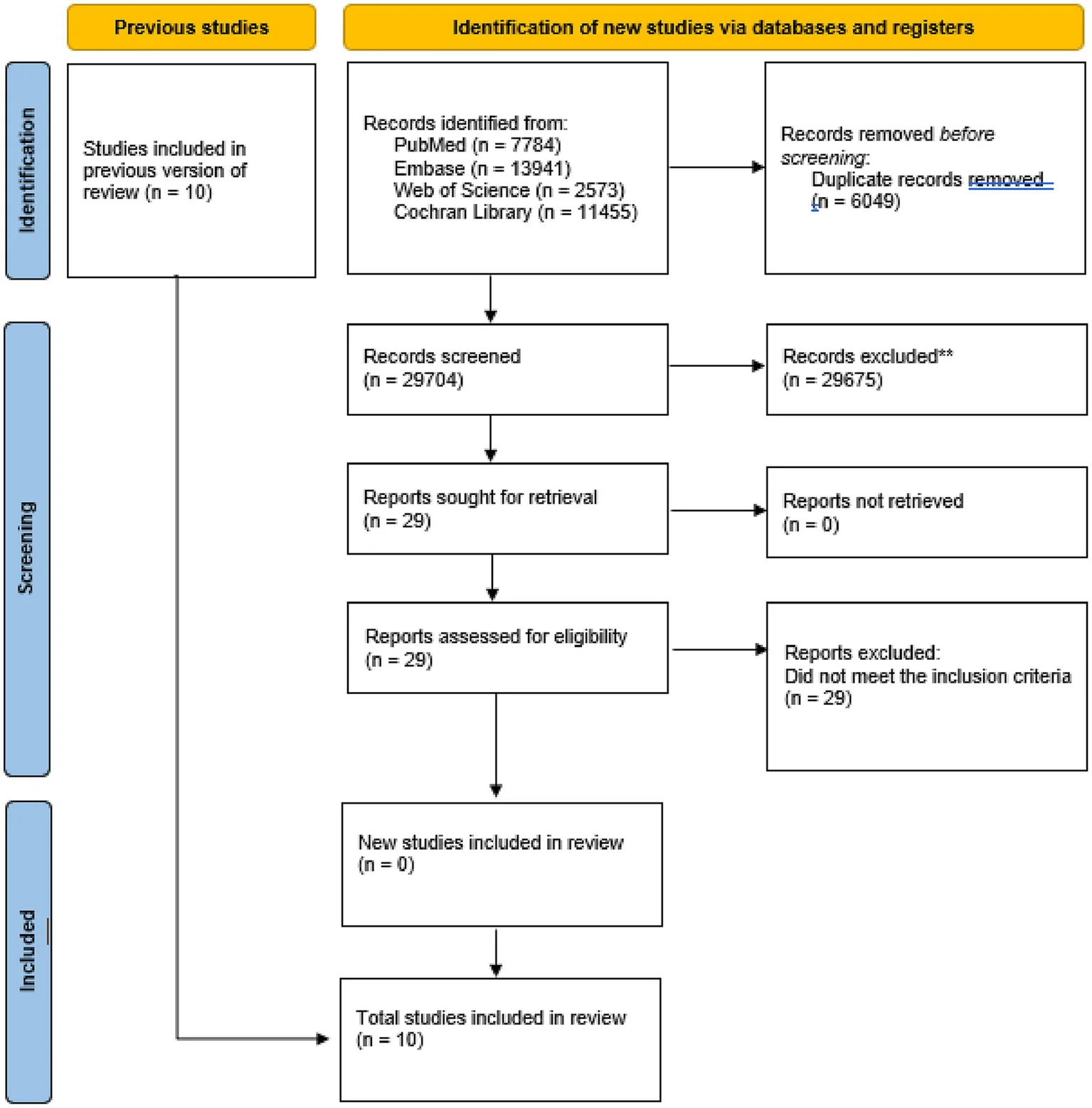
Flowchart of study selection process.

### 2.5 Data collection process

Data extraction was independently performed by two reviewers using a standardized data extraction form. Extracted information was cross-checked for accuracy, and any discrepancies were resolved through discussion; unresolved disagreements were adjudicated by a third reviewer. Study authors were not contacted for additional or missing data; analyses were based on data available in the published reports. The standardized data-extraction form was not formally pilot-tested before use.

### 2.6 Data items

The following data were extracted: first author, year of publication, country of study, sample size, participants’ age (mean ± standard deviation), years of education, type of intervention, intervention duration, intervention frequency. For global cognitive function, the mean change from baseline to post-intervention and its corresponding standard deviation were extracted or calculated for each intervention and control group. When change-score standard deviations were unavailable, they were calculated from baseline and post-intervention standard deviations according to the Cochrane Handbook for Systematic Reviews of Interventions, assuming a within-group correlation coefficient of 0.5. For BDNF and BMI, post-intervention means and standard deviations were extracted for each intervention and control group.

### 2.7 Risk of bias assessment

The risk of bias of the included randomized controlled trials was assessed using the Cochrane Risk of Bias tool version 2.0 (RoB 2.0). Two reviewers independently assessed the full texts of all eligible studies. Each study was evaluated across five domains of RoB 2.0, including bias arising from the randomization process, deviations from intended interventions, missing outcome data, measurement of the outcome, and selection of the reported result. A standardized assessment form was used to record judgments for each domain. Any discrepancies between the two reviewers were resolved through discussion or adjudication by a third reviewer until consensus was reached. The overall risk of bias for each study was categorized as “low risk,” “some concerns,” or “high risk.” Domain-level and overall judgments were determined according to the RoB 2 signaling questions and decision algorithms. An overall judgment of low risk was assigned when all domains were judged to be at low risk; some concerns was assigned when at least one domain raised some concerns but no domain was judged to be at high risk; and high risk was assigned when at least one domain was judged to be at high risk or when concerns across multiple domains substantially lowered confidence in the result. A risk-of-bias summary figure was generated to present the distribution of judgments across studies.

### 2.8 Effect measures

For global cognitive function, effect sizes were expressed as standardized mean differences (Hedges’ g) with corresponding 95% confidence intervals (CIs). A small-sample bias correction factor was applied, and effect sizes were calculated using the variance correction approach proposed by Hedges and Olkin, with corresponding standard errors appropriately adjusted. Because the included trials assessed global cognitive function using different instruments (MMSE and MoCA), Hedges’ g was used to place the effects on a common metric. Scores were oriented consistently so that higher values represented better cognitive function. For BDNF, mean differences (MDs) were used because BDNF concentrations were expressed on directly convertible concentration scales and were harmonized to a common unit before pooling; values reported in pg./mL were converted to ng/mL where necessary. Mean differences were also used for BMI because all included studies reported BMI on the same scale (kg/m^2^).

### 2.9 Synthesis methods

Statistical analyses were performed using Stata version 18.0. Between-study heterogeneity was assessed using Cochran’s Q test and quantified with the I^2^ statistic. I^2^ values of 0–25%, 25–50%, and >50% were considered to indicate low, moderate, and substantial heterogeneity, respectively. A *p*-value <0.10 for Cochran’s Q test, together with I^2^ > 50%, was considered indicative of statistically significant heterogeneity. When substantial heterogeneity was present, between-study variance was estimated using the DerSimonian–Laird method within a random-effects model; otherwise, a fixed-effect model was applied. Effect estimates were weighted using the inverse-variance method, assuming unequal between-study variances. Given the limited number of studies, exploratory univariable meta-regression analyses were conducted to examine potential associations between intervention duration, intervention frequency, and cognitive effect estimates. These analyses were considered hypothesis-generating because only eight studies contributed to the cognitive outcome. Exploratory subgroup analyses were also performed according to intervention duration (≤12 weeks vs. >12 weeks) and intervention frequency (≤2 sessions/week vs. >2 sessions/week). These cut-off points were selected based on the distribution of intervention characteristics across the included studies to permit clinically interpretable comparisons while maintaining a sufficient number of studies within each subgroup. In addition, sensitivity analyses were conducted using a leave-one-out approach to assess the robustness of the pooled results. For the cognitive outcome, an additional sensitivity analysis was performed by excluding the single study that used the MoCA and retaining only the seven studies that used the MMSE, to assess whether the pooled estimate was materially influenced by the use of different cognitive assessment instruments.

### 2.10 Reporting bias assessment

Reporting bias was assessed by evaluating the presence of selective outcome reporting across the included studies. All included studies reported the prespecified outcome measures in full, with no clear evidence of selective reporting. Overall, the risk of reporting bias was considered low.

### 2.11 Certainty assessment

The certainty of evidence for each outcome was assessed using the Grading of Recommendations Assessment, Development and Evaluation (GRADE) approach. The evaluation considered five domains: study limitations (risk of bias), inconsistency of results, indirectness, imprecision, and publication bias. The quality of evidence was categorized into four levels: high, moderate, low, and very low. Two reviewers independently performed the assessments, and any disagreements were resolved through discussion and consensus. GRADE assessments were performed separately for each outcome, taking into account the risk-of-bias judgments of the trials contributing to that specific outcome. A GRADE evidence profile was generated using GRADEpro GDT and is provided in Supplementary File 1.

## 3 Results

### 3.1 Study selection

The updated database search identified 35,753 records, including 7,784 records from PubMed, 13,941 from Embase, 2,573 from Web of Science, and 11,455 from the Cochrane Library. After removing 6,049 duplicate records, 29,704 records remained for title and abstract screening. Of these, 29,675 records were excluded, and 29 reports were sought for retrieval. All 29 reports were successfully retrieved and assessed for eligibility. After full-text assessment, all 29 reports were excluded because they did not meet the eligibility criteria, and no additional eligible studies were identified. The 10 studies [19, 20, 21, 22, 23; 24; 25; 26; 27; 28] retained from the initial search were therefore included in the final systematic review and meta-analysis. The study selection process is presented in Figure 1.

### 3.2 Study characteristics

A total of 10 studies were included, involving 446 participants. Among these, 9 studies [19, 20, 21, 23, 24, 25, 26, 27; 28] reported 51 dropouts, resulting in a final sample of 395 participants included in the analysis. All studies reported participants’ age, with mean ages ranging from 61.47 to 73.83 years. Four studies (Alves et al., 2013; Coelho-Júnior et al., 2020; Vaughan et al., 2014; Cho and Roh, 2019) reported participants’ years of education, with mean education duration ranging from 6.3 to 12.7 years.

Seven studies (Adriani et al., 2020; Alves et al., 2013; Kang et al., 2020; Coelho-Júnior et al., 2020; Farzane and Koushkie Jahromi, 2022; García-Garro et al., 2020; Cho and Roh, 2019) assessed post-intervention cognitive function using the Mini-Mental State Examination (MMSE), while one study (Smolarek et al., 2016) used the Montreal Cognitive Assessment (MoCA) for the same purpose.

The duration of physical activity interventions ranged from 8 to 24 weeks. Five studies (Adriani et al., 2020; Smolarek et al., 2016; Farzane and Koushkie Jahromi, 2022; García-Garro et al., 2020; Baharavar et al., 2025) implemented interventions lasting ≤12 weeks, while another five studies (Alves et al., 2013; Kang et al., 2020; Coelho-Júnior et al., 2020; Vaughan et al., 2014; Cho and Roh, 2019) implemented interventions lasting >12 weeks. Regarding intervention frequency, five studies (Adriani et al., 2020; Alves et al., 2013; Coelho-Júnior et al., 2020; García-Garro et al., 2020; Vaughan et al., 2014) delivered physical activity interventions ≤2 sessions per week, whereas the remaining five studies (Kang et al., 2020; Smolarek et al., 2016; Farzane and Koushkie Jahromi, 2022; Cho and Roh, 2019; Baharavar et al., 2025) delivered interventions >2 sessions per week. Across all included studies, physical activity interventions comprised Brain Gym exercises, aquatic strength training, resistance training, traditional resistance exercise, Pilates training, Taekwondo, combined circuit training, and multicomponent exercise programs. Detailed information is presented in Table 1.

**Table 1:** Characteristics of included studies.

Author and year	Country	Sample size	Education (mean ± SD), years	Age (mean ± SD), years	Intervention group intervention measures	Intervention group frequency/duration	Control group intervention measures and Duration	Outcomes
Adriani et al. (2020)	Indonesia	IG: 32 CG: 32	NR (Not reported)	IG: 65.48 ± 4.51 CG: 64.77 ± 4.31	Brain gym^®^ exercises sessions	Twice a week for 12 weeks	Not participate in any course.	Cognitive function; BDNF
Alves et al. (2013)	Brazil	IG: 14 CG: 14	IG: 6.3 ± 2.8 CG: 7.3 ± 3.1	IG: 63.9 ± 3.8 CG: 67.3 ± 5.6	Strength training	Twice a week sessions of supervised strength training for 24 weeks	Maintain their daily life activities, do not accept any intervention	Cognitive function
Kang et al. (2020)	South Korea	IG: 13 CG: 13	NR (Not reported)	IG: 73.83 ± 3.95 CG: 73.37 ± 4.19	Underwater exercise	Three times a week for 16 weeks.	Maintain their daily life activities, do not accept any intervention	Cognitive function; BDNF
Smolarek et al. (2016)	Brazil USA	IG: 29 CG: 8	NR (Not reported)	IG: 65.87 ± 5.69 CG: 65.87 ± 5.69	Strength training	3 sessions/week for 12 weeks	Maintain their daily life activities, do not accept any intervention	Cognitive function; BMI
Coelho-Júnior et al. (2020)	Brazil	IG: 15 CG: 15	IG: 7.6 ± 4.1 CG: 8.5 ± 4.3	IG: 67.0 ± 6.2 CG: 66.7 ± 4.6	Traditional resistance training	Twice a week for 22 weeks.	No exercise intervention. Contacted every 15 days for protocol adherence.	Cognitive function; BDNF
Farzane and Koushkie Jahromi (2022)	Iran	IG: 21 CG: 17	NR (Not reported)	IG: 61.47 ± 1.55 CG: 62.00 ± 1.4	Pilates training group	3 sessions/week for 8 weeks	Maintain their daily life activities, do not accept any intervention	Cognitive function; BMI
García-Garro et al. (2020)	Spain	IG: 55 CG: 55	NR (Not reported)	IG: 69.98 ± 7.83 CG: 66.79 ± 10.14	Clinical Pilates movement	60 min twice a week for 12 weeks	Maintain their daily life activities, do not accept any intervention	Cognitive function
Cho and Roh (2019)	South Korea	IG: 20 CG: 20	IG: 11.33 ± 2.47 CG: 11.37 ± 2.41	IG: 68.89 ± 4.16 CG: 69.00 ± 4.41	Taekwondo	Five times a week for a total of 16 weeks.	Maintain their daily life activities, do not accept any intervention	Cognitive function; BDNF
Baharavar et al. (2025)	Iran	IG: 12 CG: 12	NR (Not reported)	IG: 65 ± 3 CG: 64 ± 3	Mixed cycle training	2 times a week for the first 2 weeks and 3 times a week for the 3rd to 8th weeks	Maintain their daily life activities, do not accept any intervention	Body Mass Index (BMI)
Vaughan et al. (2014)	Australia	IG: 25 CG: 24	IG: 12.3 ± 2.9 CG: 12.7 ± 3.5	IG: 69 ± 3.1 CG: 68.8 ± 3.5	Multi-modal motion plan	Twice weekly for a total of 16 weeks	Maintain their daily life activities, do not accept any intervention	BDNF

### 3.3 Risk of bias in included studies

Methodological quality of the 10 included randomized controlled trials was assessed using the Cochrane Risk of Bias tool version 2.0 (RoB 2.0). The assessment covered five domains: bias arising from the randomization process, deviations from intended interventions, missing outcome data, measurement of the outcome, and selection of the reported result. An overall risk-of-bias judgment was generated for each study based on domain-level assessments. Study-level risk-of-bias judgments and the corresponding summary proportions are presented in Figure 2.

**Figure 2:**
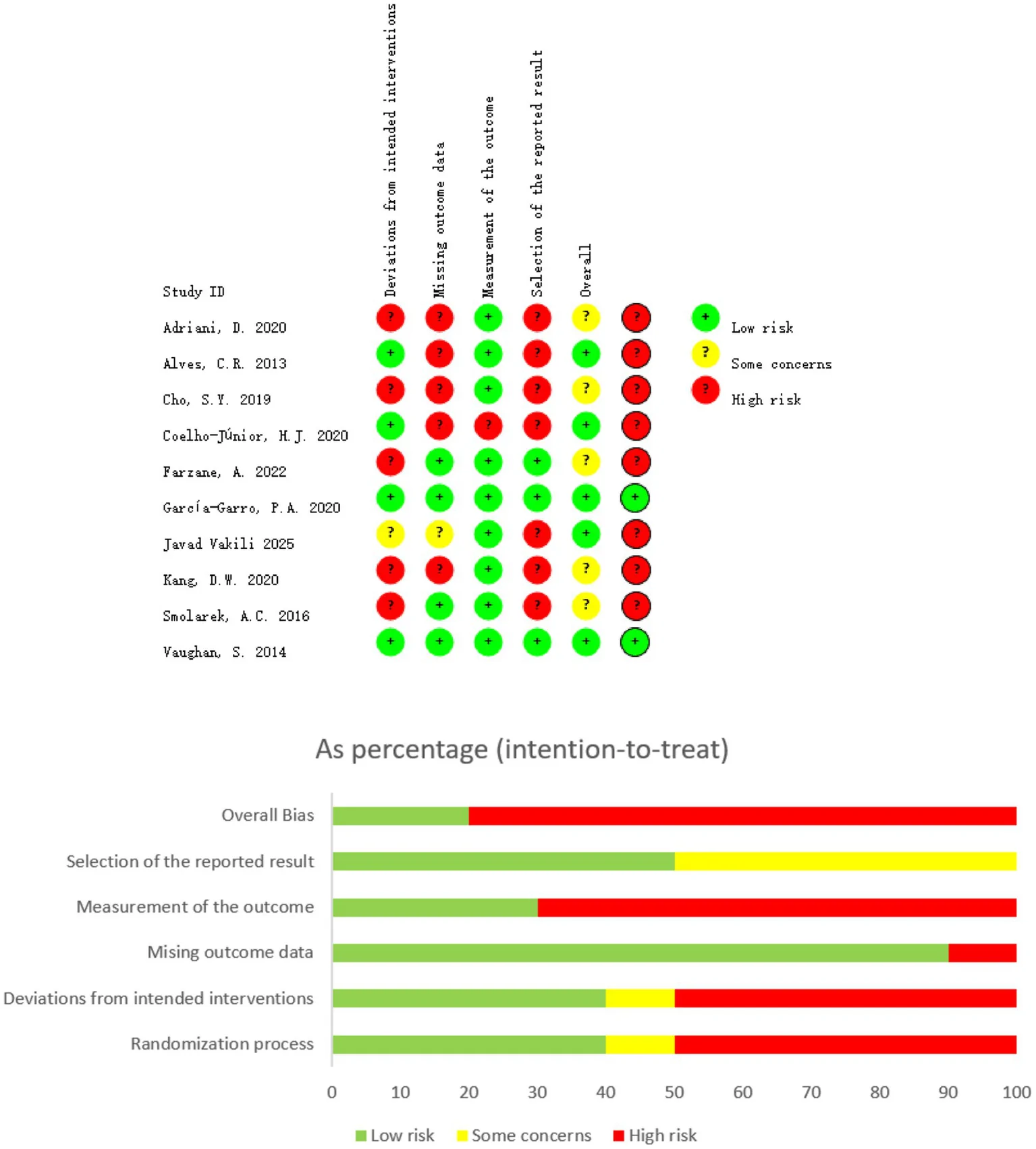
Risk-of-bias assessment of the included randomized controlled trials using the Cochrane RoB 2 tool. The upper panel presents study-level judgments across the five RoB 2 domains and overall risk of bias, and the lower panel presents the percentage distribution of risk-of-bias judgments across domains.

For bias arising from the randomization process, four studies (40%) were judged to be at low risk, one study (10%) raised some concerns, and five studies (50%) were judged to be at high risk. For bias due to deviations from intended interventions, four studies (40%) were judged to be at low risk, one study (10%) raised some concerns, and five studies (50%) were judged to be at high risk. Bias due to missing outcome data was generally less concerning, with nine studies (90%) judged to be at low risk and one study (10%) at high risk. In contrast, bias in measurement of the outcome represented a major methodological concern: three studies (30%) were judged to be at low risk, whereas seven studies (70%) were judged to be at high risk. For bias in selection of the reported result, five studies (50%) were judged to be at low risk and five studies (50%) raised some concerns, with no studies judged to be at high risk in this domain. Overall, two studies (20%) were judged to be at low risk of bias, whereas eight studies (80%) were judged to be at high overall risk of bias. No study received an overall judgment of some concerns. These findings indicate substantial methodological limitations across the included evidence base, particularly in the randomization process, deviations from intended interventions, and measurement of outcomes. These limitations should be considered when interpreting the pooled effect estimates.

Detailed results are presented in Figure 2.

### 3.4 Synthesis of results

#### 3.4.1 Global cognitive function

Eight trials (Adriani et al., 2020; Alves et al., 2013; Kang et al., 2020; Smolarek et al., 2016; Coelho-Júnior et al., 2020; Farzane and Koushkie Jahromi, 2022; García-Garro et al., 2020; Cho and Roh, 2019) involving 332 participants evaluated the effects of physical activity on global cognitive function. Seven trials used the MMSE and one trial used the MoCA. Because these instruments assess global cognitive function using different measurement scales, the effects were standardized and synthesized using Hedges’ g. The pooled analysis showed a statistically significant effect on global cognitive function (Hedges’ g = 0.63, 95% CI: 0.12–1.13, *p* = 0.02). Detailed results are presented in Figure 3.

**Figure 3:**
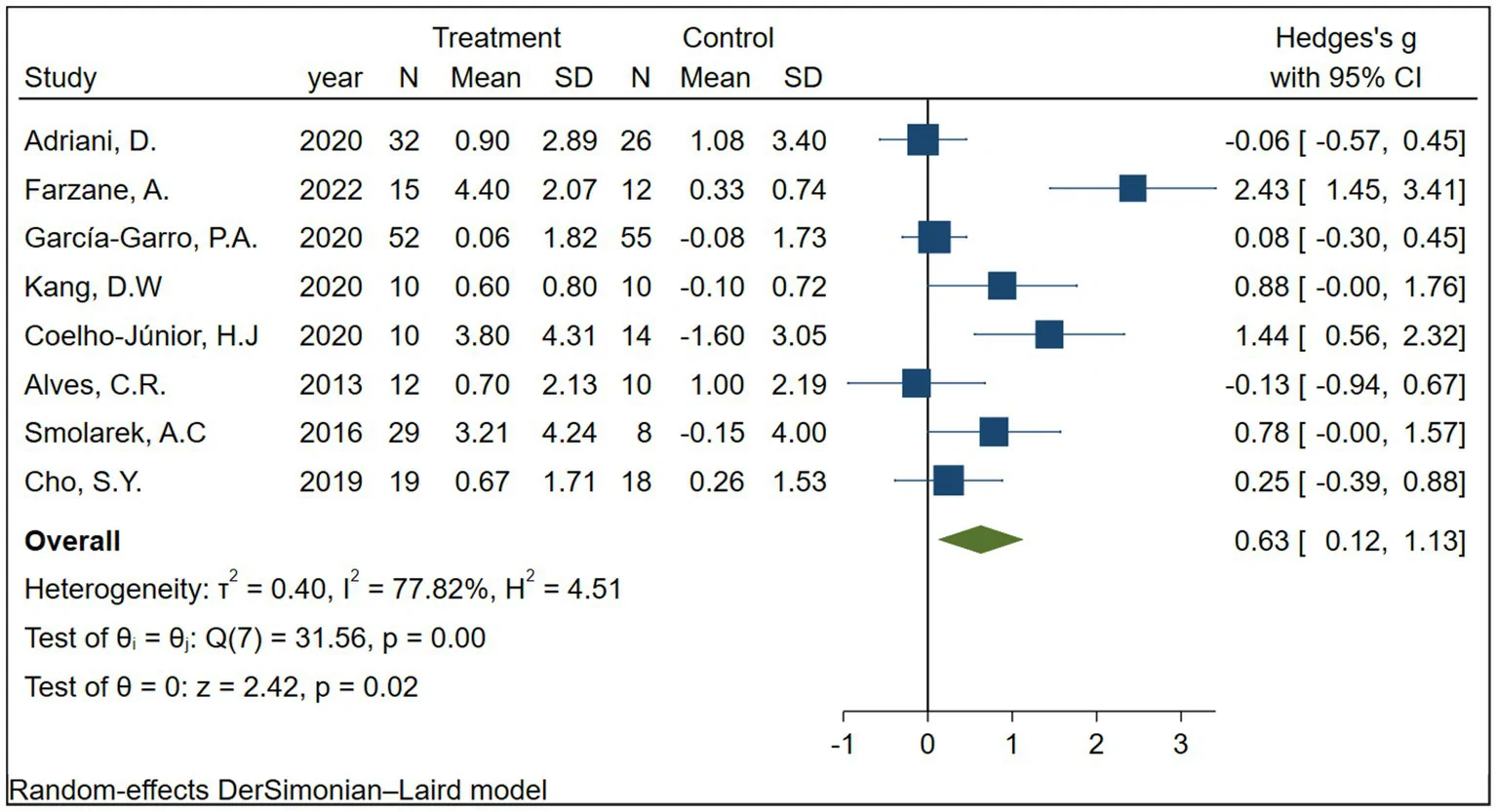
Forest plot of the effects of physical activity on global cognitive function in healthy older women. Means and standard deviations represent changes from baseline to post-intervention.

#### 3.4.2 BDNF

Five trials (Adriani et al., 2020; Kang et al., 2020; Coelho-Júnior et al., 2020; Vaughan et al., 2014; Cho and Roh, 2019) involving 188 participants examined the effects of physical activity on brain-derived neurotrophic factor (BDNF) levels in older women. Compared with the control group, physical activity was associated with a statistically significant increase in BDNF levels (MD = 2.43, 95% CI: 0.57–4.28, *p* = 0.01). Detailed results are presented in Figure 4.

**Figure 4:**
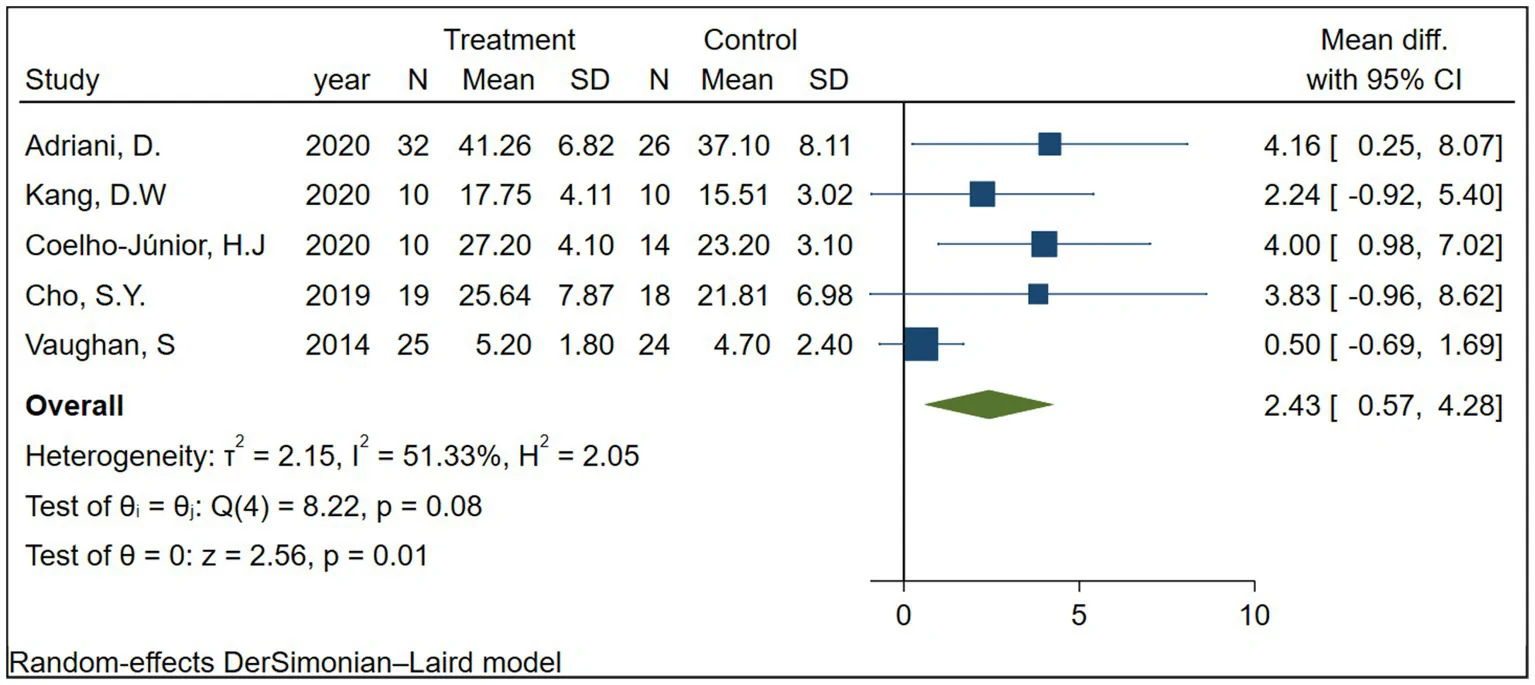
Forest plot of the effects of physical activity on brain-derived neurotrophic factor (BDNF) levels in healthy older women. Means and standard deviations represent post-intervention BDNF levels (ng/mL).

#### 3.4.3 BMI

Three trials (Smolarek et al., 2016; Farzane and Koushkie Jahromi, 2022; Baharavar et al., 2025) comprising 87 participants evaluated the effects of physical activity on body mass index (BMI) in older women. No statistically significant difference in BMI was observed between the physical activity and control groups (MD = 0.63, 95% CI: −1.12–2.37, *p* = 0.48). Detailed results are presented in Figure 5.

**Figure 5:**
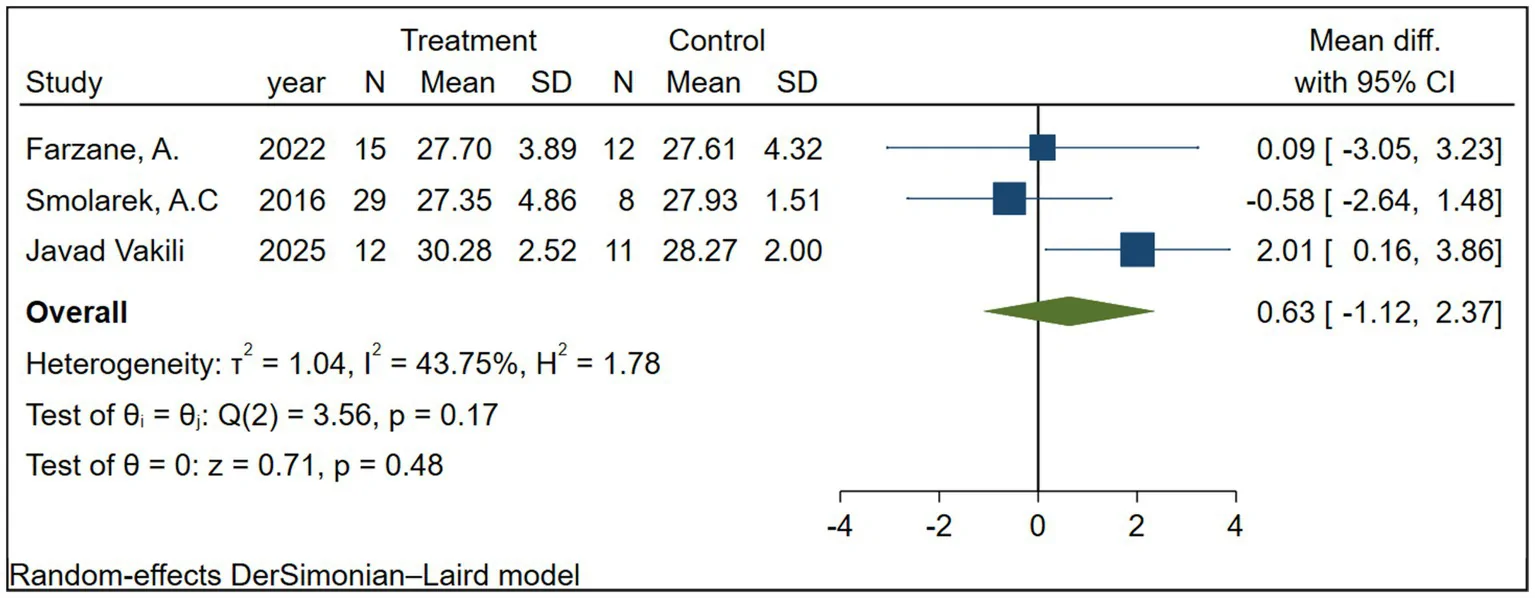
Forest plot of the effects of physical activity on body mass index (BMI) in healthy older women. Means and standard deviations represent post-intervention BMI values (kg/m²).

### 3.5 Meta-regression

Meta-regression analyses were conducted using total intervention duration and intervention frequency as covariates. No statistically significant association was detected between effect size and either total intervention duration (coefficient = −0.155, *p* = 0.782) or intervention frequency (coefficient = 0.749, *p* = 0.216). Given the small number of studies, these null findings should be interpreted cautiously and do not exclude potential moderating effects. Detailed results are presented in Table 2.

**Table 2:** Meta-regression results for the effects of total intervention duration and intervention frequency on global cognitive function in healthy older women.

Outcome	Variable	Coef.	*T*	*P* > |*t*|	[95% CI]
Cognitive function	Duration of intervention	−0.155	−0.29	0.782	−1.512, 1.203
	Frequency of intervention	0.749	1.42	0.216	−0.610, 2.107

### 3.6 Subgroup analyses

To further investigate potential moderators of the effects of physical activity on cognitive function in healthy older women, subgroup analyses were performed according to total intervention duration (≤12 weeks vs. >12 weeks) and weekly intervention frequency (≤2 sessions/week vs. >2 sessions/week).

Total intervention duration: Physical activity interventions lasting ≤12 weeks did not yield a statistically significant effect on cognitive function in older women (Hedges’ g = 0.71, 95% CI: −0.13–1.54, *p* = 0.10). Similarly, interventions exceeding 12 weeks showed no statistically significant improvement (Hedges’ g = 0.57, 95% CI: −0.08–1.22, *p* = 0.08). The between-subgroup difference was not statistically significant (*p* = 0.82), indicating no evidence that intervention duration significantly modified the effect of physical activity on cognitive function.

Intervention frequency: Physical activity performed at a frequency of ≤2 sessions per week showed no statistically significant pooled effect on cognitive function (Hedges’ g = 0.25, 95% CI: −0.28–0.79, *p* = 0.36), whereas the pooled effect was statistically significant for interventions performed >2 sessions per week (Hedges’ g = 1.03, 95% CI: 0.18–1.89, *p* = 0.02). However, the between-subgroup difference was not statistically significant (*p* = 0.12), providing no evidence that intervention frequency significantly modified the effect of physical activity on cognitive function. Detailed results are presented in Table 3.

**Table 3:** Subgroup analyses of the effects of physical activity on cognitive function in healthy older women stratified by total intervention duration and intervention frequency.

Subgroup variable	Subgroup	Participants (I/C)	Hedges’ g	95% CI	*P* (Overall effect)	*P* (Heterogeneity)	I^2^, %	*P* (group difference)
Total duration of physical activity intervention	≤12 weeks	(128/101)	0.71	[−0.13, 1.54]	0.10	<0.001	86.82%	0.82
	>12 weeks	(51/52)	0.57	[−0.08, 1.22]	0.08	0.05	62.69%	
	Total	(179/153)	0.63	[0.12, 1.13]	0.02	<0.001	77.82%	
Frequency of physical activity intervention	≤2 times a week	(106/105)	0.25	[−0.28, 0.79]	0.36	0.02	68.24%	0.12
	>2 times a week	(73/48)	1.03	[0.18, 1.89]	0.02	<0.005	77.73%	
	Total	(179/153)	0.63	[0.12, 1.13]	0.02	<0.001	77.82%	

### 3.7 Sensitivity analysis

Sensitivity analyses were conducted for all outcome measures included in this meta-analysis by sequentially omitting each individual study. The results demonstrated that no single study shifted the pooled effect estimate beyond the 95% confidence interval of the overall mean difference, indicating that the findings were robust. For the cognitive outcome, an additional sensitivity analysis was conducted by excluding the single study that used the MoCA and retaining only the seven studies that used the MMSE. The pooled effect remained statistically significant and was similar in magnitude to the primary analysis (Hedges’ g = 0.60, 95% CI: 0.04–1.16, *p* = 0.03). Substantial heterogeneity remained (I^2^ = 78.41%; Q (6) = 27.80, *p* < 0.001). These findings indicate that exclusion of the MoCA-based study did not materially alter the pooled effect estimate, although substantial between-study heterogeneity persisted. An additional sensitivity analysis excluding studies judged to be at high overall risk of bias was considered. However, such an analysis was not feasible because exclusion of high-risk studies would leave an insufficient number of studies for meta-analytic pooling within each outcome. Therefore, the influence of high-risk studies could not be formally evaluated through a risk-of-bias-based sensitivity analysis. The leave-one-out sensitivity analyses are presented in Figure 6, whereas the sensitivity analysis restricted to MMSE-based studies is presented in Supplementary Figure S1.

**Figure 6:**
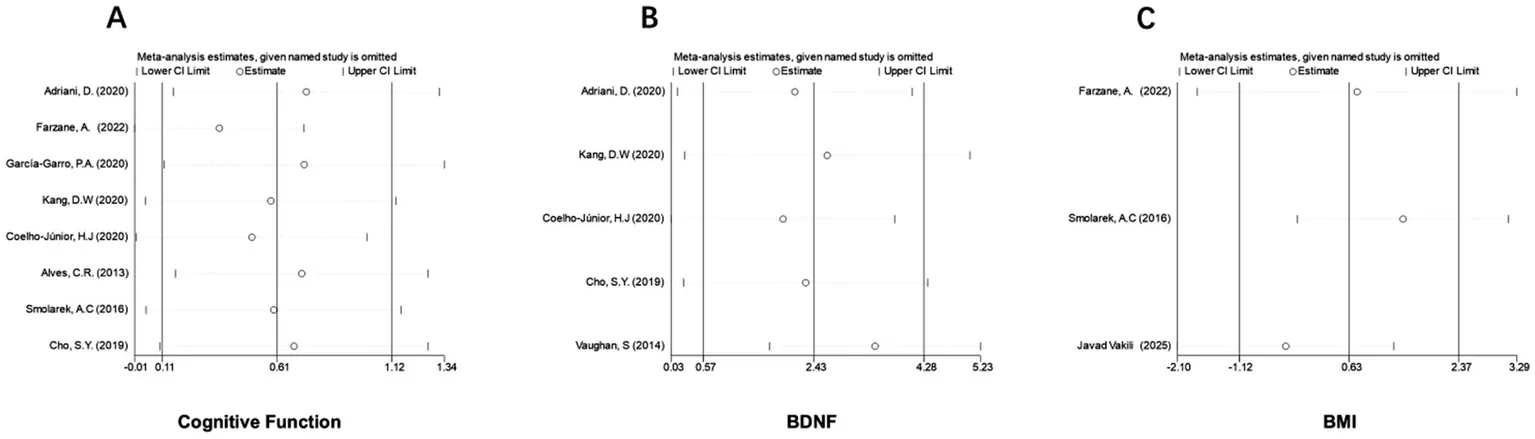
Sensitivity analyses using the leave-one-out method. **(A)** Cognitive function; **(B)** Brain-derived neurotrophic factor; **(C)** Body mass index.

### 3.8 Certainty of evidence

The certainty of evidence for each outcome was appraised using the Grading of Recommendations, Assessment, Development and Evaluations (GRADE) approach. For global cognitive function, the certainty of evidence was rated as very low. The evidence was downgraded by two levels for risk of bias because most trials contributing to this outcome were judged to be at high overall risk of bias, with important concerns related to the randomization process, deviations from intended interventions, and measurement of the outcome. The evidence was further downgraded by one level for inconsistency because substantial between-study heterogeneity was observed (I^2^ = 77.82%).

For BDNF, the certainty of evidence was also rated as very low. The evidence was downgraded by two levels for risk of bias because most contributing trials were judged to be at high overall risk of bias, and by one level for inconsistency because moderate-to-substantial heterogeneity was observed (I^2^ = 51.33%) and its source could not be fully explained.

For BMI, the certainty of evidence was rated as very low. All three contributing trials were judged to be at high overall risk of bias, resulting in a two-level downgrade for risk of bias. The evidence was additionally downgraded by one level for imprecision because only 87 participants contributed to the analysis and the 95% confidence interval (−1.12 to 2.37) crossed the null value and included effects in both directions. No serious indirectness was identified for any of the three outcomes. Detailed GRADE assessments are presented in Supplementary File 1.

## 4 Discussion

This meta-analysis examined the effects of physical activity on global cognitive function, brain-derived neurotrophic factor (BDNF), and body mass index (BMI) in healthy older women. The pooled analyses showed statistically significant effects on global cognitive function and BDNF levels; however, the certainty of evidence for these findings was very low. The risk-of-bias assessment revealed substantial methodological limitations in the included evidence base, with eight of ten trials judged to be at high overall risk of bias. High-risk judgments were particularly frequent in the domains of outcome measurement, the randomization process, and deviations from intended interventions. Blinding of participants and intervention personnel is often impractical in exercise trials; however, lack of blinding alone does not automatically constitute a high risk of bias under RoB 2. Rather, the risk of bias depends on whether knowledge of intervention assignment led to deviations from the intended interventions or influenced outcome measurement. These limitations reduce confidence in the pooled estimates and may have influenced the magnitude and precision of the observed effects. Therefore, statistically significant pooled effects should not be interpreted as definitive evidence of intervention efficacy, particularly given the limited certainty of the available evidence.

To explore potential effect modification in cognitive function outcomes, meta-regression analyses were performed using total intervention duration and weekly intervention frequency as covariates. No statistically significant associations were detected for either covariate. Consistently, subgroup analyses showed no statistically significant between-subgroup differences according to intervention duration (*p* = 0.82) or intervention frequency (*p* = 0.12). Although the pooled effect was statistically significant among interventions performed more than twice per week (Hedges’ g = 1.03, 95% CI: 0.18–1.89, *p* = 0.02), the formal test for subgroup differences was not statistically significant. Therefore, this finding should not be interpreted as evidence that a frequency of more than two sessions per week is superior to a lower intervention frequency. Given the small number of studies within each subgroup, these analyses have limited statistical power and should be regarded as exploratory.

Substantial between-study heterogeneity was observed for global cognitive function (I^2^ = 77.82%). Although intervention duration and weekly frequency did not significantly explain this heterogeneity in the subgroup and meta-regression analyses, several other sources may have contributed. The included trials employed diverse exercise modalities, including Brain Gym, aquatic exercise, resistance training, Pilates, Taekwondo, and multicomponent exercise, which may differ in their physiological and cognitive demands. In addition, variation in cognitive assessment instruments, participant educational and cultural backgrounds, and other study-level characteristics may have contributed to differences in observed effect sizes. Notably, exclusion of the single MoCA-based study did not reduce heterogeneity (I^2^ = 78.41%), suggesting that differences in cognitive assessment instruments alone were unlikely to account for the observed heterogeneity. Because only eight studies contributed to the cognitive analysis, the available data were insufficient to reliably investigate multiple additional moderators. Therefore, the sources of heterogeneity remain only partially explained, and the pooled cognitive effect should be interpreted with caution. The present findings are broadly consistent with the existing body of meta-analytic evidence, although the specific population examined differs. A substantial literature has demonstrated that exercise significantly improves global cognitive function in older adults. For instance, Zhang et al. (2022) reported that aerobic exercise yielded significant cognitive benefits in older populations. Another meta-analysis found that different modalities of physical activity can selectively enhance specific cognitive domains, including executive function, memory, and processing speed (Kelly et al., 2014). Furthermore, Sanders et al. (2019) and Wayne et al. (2014) demonstrated that physical activity confers mild-to-moderate improvements in global cognitive function among older adults with cognitive impairment, including those with mild cognitive impairment (MCI) and Alzheimer’s disease (AD). However, few studies have specifically investigated the effects of physical activity interventions on global cognitive function in healthy older women. The present meta-analysis addresses this gap and provides novel evidence to inform our understanding of this relationship.

A distinguishing feature of the present meta-analysis is the strict inclusion of randomized controlled trials enrolling exclusively healthy older women. This population-specific focus is supported by prior evidence suggesting that biological sex may moderate the cognitive benefits of exercise. Barha et al. (2017) found that the effects of aerobic exercise on executive function were larger in studies with a higher proportion of female participants, indicating that women may exhibit greater cognitive responsiveness to specific modalities of physical activity.

The present meta-analysis identified statistically significant pooled effects on cognitive function and BDNF levels in healthy older women; however, these findings should be interpreted cautiously given the very low certainty of evidence. The sensitivity analysis restricted to the seven MMSE-based trials yielded an effect estimate similar to that of the primary analysis, suggesting that the pooled cognitive effect was not materially driven by the single MoCA-based study. However, substantial heterogeneity persisted after exclusion of the MoCA study (I^2^ = 78.41%), indicating that differences in cognitive assessment instruments alone are unlikely to account for the observed between-study heterogeneity. These parallel improvements lend partial support to the hypothesis proposed by Farrukh et al. (2023) that physical activity may positively influence cognition and neural health through multiple molecular pathways (Seyfizadeh et al., 2025). Consistent with this, a trial involving older women reported that a 12-week combined aerobic and resistance exercise program improved both cognitive function and BDNF expression (Byun and Kang, 2016). Mechanistically, exercise-induced elevations in BDNF have been attributed to cellular signaling cascades involving tropomyosin receptor kinase B (TrkB), insulin-like growth factor-1 (IGF-1), and vascular endothelial growth factor (VEGF) (Jiménez-Maldonado et al., 2014). Moreover, increased BDNF expression is considered critical for neuronal survival, synaptic plasticity, and hippocampal neurogenesis in adulthood (Azarfarin et al., 2025a; Azarfarin et al., 2025b). Importantly, however, the present study did not directly examine the relationship between changes in BDNF and improvements in cognitive function, and whether BDNF elevation serves as a mediating mechanism underlying exercise-related cognitive benefits remains inconclusive. Notably, Farrukh et al. (2023) reported that while exercise produced a clear increase in BDNF among older women, the accompanying improvement in MMSE scores did not reach statistical significance (Cohen’s d = 0.17, *p* = 0.73). This discordance underscores the complexity of the BDNF–cognition relationship and cautions against simplistic linear inferences. That both BDNF and cognitive function were significantly improved in the present meta-analysis suggests that physical activity may exert cognitive benefits through multiple pathways, of which BDNF-mediated signaling represents one contributory mechanism.

Compared with the control group, physical activity did not produce a statistically significant change in BMI among healthy older women (MD = 0.63, 95% CI: −1.12–2.37, *p* = 0.48). This null finding may be attributable to several factors. First, the analysis included only three trials with a cumulative sample of 87 participants, resulting in insufficient statistical power to detect a potentially meaningful effect (Zang et al., 2020). Second, the included studies employed heterogeneous exercise modalities, and evidence suggests that different types of exercise exert differential effects on body composition — aerobic and resistance training, for example, do not produce equivalent changes in BMI (Villareal et al., 2017). Third, the intervention durations across included studies ranged from 8 to 24 weeks, representing short-to-medium-term exposure. Prior research indicates that exercise interventions of this duration typically do not yield significant reductions in BMI, as the predominant physiological response during this phase is body composition remodeling — characterized by concurrent fat mass loss and lean mass gain — rather than measurable changes in overall body weight or BMI (Villareal et al., 2017). Taken together, these considerations suggest that BMI may lack the sensitivity to capture the effects of short-to-medium-term exercise interventions in older women. Future studies should consider adopting more refined body composition measures, such as body fat percentage, waist circumference, or lean muscle mass, as primary or co-primary endpoints. It is worth noting that the direction of the pooled effect estimate (MD = +0.63) suggests a slight, albeit non-significant, increase in BMI in the intervention group relative to controls. This directionality is consistent with the body composition remodeling hypothesis: concurrent gains in lean muscle mass may offset or exceed reductions in fat mass, leading to a stable or marginally elevated BMI despite favorable changes in body composition. This observation further supports the argument that BMI may be an insensitive metric for capturing the physiological effects of exercise interventions in older women.

The certainty of evidence for all three outcomes was rated as very low using the GRADE approach. For global cognitive function and BDNF, the principal reasons for downgrading were the high risk of bias among contributing trials and between-study inconsistency. For BMI, the evidence was downgraded primarily because all contributing trials were judged to be at high overall risk of bias and because the pooled estimate was imprecise, with a wide confidence interval crossing the null. These very-low-certainty ratings indicate that confidence in the pooled effect estimates is limited and that the true effects may differ substantially from the observed estimates. Accordingly, the statistically significant pooled findings for cognitive function and BDNF should be regarded as suggestive rather than definitive evidence of benefit.

Several strengths of this meta-analysis merit consideration. First, the analysis was restricted exclusively to randomized controlled trials, enhancing the internal validity of the pooled estimates. Second, by focusing specifically on healthy older women, this review addresses a population that has been underrepresented in the existing literature and elucidates the effects of physical activity — a modifiable lifestyle factor — on global cognitive function in this demographic.

Despite the favorable findings regarding the effects of physical activity on global cognitive function in older women, several limitations of this meta-analysis should be acknowledged. First, of the ten included trials, only eight reported cognitive function outcomes, limiting the volume of evidence available for the primary analysis. Furthermore, the subgroup analyses were based on only eight studies, with four studies in each subgroup, which limited the statistical power to detect between-subgroup differences. Accordingly, these subgroup findings should be regarded as exploratory and interpreted with caution. Second, global cognitive function was assessed using the MMSE in seven studies and the MoCA in one study. Although standardized mean differences were used to place effects measured by these instruments on a common metric, the MMSE and MoCA differ in their sensitivity and cognitive domain coverage; therefore, some measurement heterogeneity may remain despite statistical standardization. In addition, cognitive screening scores may be influenced by factors such as language proficiency, literacy level, cultural background, and years of formal education (Espino et al., 2001; Escobar et al., 1986).

Third, only three studies contributed BMI data, and the sample sizes were unevenly distributed between the intervention (*n* = 56) and control (*n* = 31) groups, resulting in insufficient statistical power to draw reliable conclusions regarding the effects of physical activity on BMI. In addition, reporting of BDNF specimen type, assay method, and sampling time was not uniform across the included trials; therefore, these methodological characteristics could not be examined as separate moderators and may have limited the comparability of the pooled BDNF estimate. Fourth, formal assessment of publication bias using funnel plots or Egger’s regression test was not feasible owing to the limited number of studies included for each outcome (*n* < 10), which falls below the recommended threshold for reliable detection of funnel plot asymmetry. The possibility of publication bias therefore cannot be excluded. Fifth, the included studies employed heterogeneous exercise modalities (e.g., aerobic training, resistance training, Pilates, Taekwondo), which limits the ability to determine optimal exercise prescriptions. Sixth, no included trial assessed the long-term maintenance of cognitive benefits beyond the intervention period; whether the observed improvements persist after cessation of physical activity remains unknown. Seventh, the MMSE is known to exhibit ceiling effects in cognitively healthy populations, potentially attenuating the magnitude of detectable cognitive improvements among participants with near-maximum baseline scores. In light of these limitations, future research should prioritize larger sample sizes and employ more comprehensive and culturally robust cognitive assessment batteries — such as the Montreal Cognitive Assessment (MoCA) or domain-specific neuropsychological tests — to more accurately characterize the cognitive effects of physical activity in healthy older women. Eighth, when change-score standard deviations were unavailable, a pre–post correlation coefficient of 0.5 was assumed. Because sensitivity analyses using alternative correlation coefficients were not performed, uncertainty associated with this assumption cannot be excluded. Ninth, the included trials enrolled relatively small samples of healthy older women from a limited number of countries. Therefore, the findings may not be generalizable to older men, adults with cognitive impairment, or older women with substantial comorbidities.

## 5 Conclusion

This meta-analysis synthesized evidence from ten randomized controlled trials encompassing 446 participants. Very-low-certainty evidence suggests that physical activity may improve global cognitive test performance and increase BDNF levels in healthy older women, whereas no statistically significant effect on BMI was observed. However, substantial between-study heterogeneity was present in the global cognitive function analysis (I^2^ = 77.82%), and confidence in the findings was limited by the high risk of bias, inconsistency for global cognitive function and BDNF, and imprecision for BMI. Therefore, the pooled findings should be interpreted cautiously and should not be considered definitive evidence of efficacy or evidence that physical activity prevents dementia. Formal tests for subgroup differences showed no statistically significant effect modification according to intervention duration or frequency. Further well-designed randomized controlled trials with rigorous methodology, larger sample sizes, standardized outcome measures, and longer follow-up periods are needed to confirm these findings.

## Data Availability

The original contributions presented in the study are included in the article/Supplementary material, further inquiries can be directed to the corresponding author.
