# Supplementary material for: Effects of physical activity on cognitive function in healthy older women: a systematic review and meta-analysis
Source: Front Aging Neurosci. 2026 Oct 15;18:1928056. doi: 10.3389/fnagi.2026.1928056 (PMC13716729; doi:10.3389/fnagi.2026.1928056)
Supplement: Supplementary file 1 [file Supplementary_file_1.docx]

Supplementary Figure S1. Sensitivity analysis of global cognitive function restricted to studies using the Mini-Mental State Examination (MMSE).

The single trial using the Montreal Cognitive Assessment (MoCA) was excluded, leaving seven trials using the MMSE. The pooled effect was estimated using a random-effects DerSimonian–Laird model (Hedges’ g = 0.60, 95% CI: 0.04–1.16, P = 0.03). Substantial between-study heterogeneity remained (I² = 78.41%; Q(6) = 27.80, P < 0.001). MMSE, Mini-Mental State Examination; MoCA, Montreal Cognitive Assessment; SD, standard deviation; CI, confidence interval.
