# Supplementary material for: Effects of physical activity on cognitive function in healthy older women: a systematic review and meta-analysis
Source: Front Aging Neurosci. 2026 Oct 15;18:1928056. doi: 10.3389/fnagi.2026.1928056 (PMC13716729; doi:10.3389/fnagi.2026.1928056)
Supplement: Supplementary file 3 [file Table_2.DOCX]

Supplementary Table S1 Full Search Strategies

The databases were searched from inception to 26 September 2026 without language restrictions. The search strategies below are reproduced in database-specific syntax as run.

# **Embase**

Platform: Embase.com | Date searched: 26 September 2026
Records retrieved: 13,941

| **Line** | **Search strategy** |
| --- | --- |
| **#1** | 'biometric exercise'/exp OR 'biometric exercise' OR 'effort'/exp OR 'effort' OR 'exercise capacity'/exp OR 'exercise capacity' OR 'exercise performance'/exp OR 'exercise performance' OR 'exercise training'/exp OR 'exercise training' OR 'exertion'/exp OR 'exertion' OR 'fitness training'/exp OR 'fitness training' OR 'fitness workout'/exp OR 'fitness workout' OR 'physical conditioning, human'/exp OR 'physical conditioning, human' OR 'physical effort'/exp OR 'physical effort' OR 'physical exercise'/exp OR 'physical exercise' OR 'physical exertion'/exp OR 'physical exertion' OR 'physical work-out'/exp OR 'physical work-out' OR 'physical workout'/exp OR 'physical workout' OR 'exercise'/exp OR 'exercise' |
| **#2** | 'cognitive accessibility'/exp OR 'cognitive accessibility' OR 'cognitive balance'/exp OR 'cognitive balance' OR 'cognitive dissonance'/exp OR 'cognitive dissonance' OR 'cognitive function'/exp OR 'cognitive function' OR 'cognitive functioning'/exp OR 'cognitive functioning' OR 'cognitive structure'/exp OR 'cognitive structure' OR 'cognitive symptoms'/exp OR 'cognitive symptoms' OR 'cognitive task'/exp OR 'cognitive task' OR 'cognitive thinking'/exp OR 'cognitive thinking' OR 'neurobehavioural manifestations'/exp OR 'neurobehavioural manifestations' OR 'volition'/exp OR 'volition' OR 'cognition'/exp OR 'cognition' |
| **#3** | 'females'/exp OR 'females' OR 'woman'/exp OR 'woman' OR 'women'/exp OR 'women' OR 'female'/exp OR 'female' |
| **#4** | #1 AND #2 |
| **#5** | #3 AND #4 |
| **#6** | 'controlled trial, randomized'/exp OR 'controlled trial, randomized' OR 'randomised controlled study'/exp OR 'randomised controlled study' OR 'randomised controlled trial'/exp OR 'randomised controlled trial' OR 'randomized controlled study'/exp OR 'randomized controlled study' OR 'trial, randomized controlled'/exp OR 'trial, randomized controlled' OR 'randomized controlled trial'/exp OR 'randomized controlled trial' |
| **#7** | #5 AND #6 |

# **PubMed**

Platform: NCBI PubMed | Date searched: 26 September 2026
Records retrieved: 7,784

| **Line** | **Search strategy** |
| --- | --- |
| **#1** | ("Exercise"[Mesh] OR "Exercise Therapy"[Mesh] OR "Motor Activity"[Mesh] OR "Physical Exertion"[Mesh] OR "Exercise Tolerance"[Mesh] OR "Physical Fitness"[Mesh] OR exercis*[Title/Abstract] OR "physical activit*"[Title/Abstract] OR "exercise train*"[Title/Abstract] OR "physical train*"[Title/Abstract] OR exertion[Title/Abstract] OR "physical effort"[Title/Abstract] OR "physical workout*"[Title/Abstract] OR fitness[Title/Abstract]) AND ("Cognition"[Mesh] OR "Cognitive Dysfunction"[Mesh] OR "Neuropsychological Tests"[Mesh] OR cognit*[Title/Abstract] OR "cognitive function*"[Title/Abstract] OR "cognitive task*"[Title/Abstract] OR "cognitive symptom*"[Title/Abstract] OR "neurobehavioral manifestation*"[Title/Abstract] OR volition[Title/Abstract]) AND ("Female"[Mesh] OR "Women"[Mesh] OR female*[Title/Abstract] OR woman[Title/Abstract] OR women[Title/Abstract]) AND (randomized controlled trial[Publication Type] OR controlled clinical trial[Publication Type] OR random*[Title/Abstract] OR trial[Title/Abstract]) |

# **Cochrane Central Register of Controlled Trials**

Platform: Cochrane Library | Date searched: 26 September 2026
Records retrieved: 11,455 Trials

| **Line** | **Search strategy** |
| --- | --- |
| **#1** | MeSH descriptor: [Exercise] explode all trees |
| **#2** | (exercis* OR "physical activity" OR "physical activities" OR "exercise training" OR "physical training" OR exertion OR "physical effort" OR "physical workout" OR "physical workouts" OR fitness):ti,ab,kw |
| **#3** | #1 OR #2 |
| **#4** | MeSH descriptor: [Cognition] explode all trees |
| **#5** | (cognit* OR "cognitive function" OR "cognitive functions" OR "cognitive functioning" OR "cognitive task" OR "cognitive tasks" OR "cognitive symptom" OR "cognitive symptoms" OR "cognitive thinking" OR "neurobehavioral manifestations" OR "neurobehavioural manifestations" OR volition):ti,ab,kw |
| **#6** | #4 OR #5 |
| **#7** | MeSH descriptor: [Women] explode all trees |
| **#8** | (female* OR woman OR women):ti,ab,kw |
| **#9** | #7 OR #8 |
| **#10** | #3 AND #6 AND #9 |

# **Web of Science Core Collection**

Platform: Clarivate Web of Science | Date searched: 26 September 2026
Records retrieved: 2,573

| **Line** | **Search strategy** |
| --- | --- |
| **#1** | TS=( ( ((older OR elderly OR aged) NEAR/0 (woman OR women OR female*)) OR (postmenopaus* NEAR/0 (woman OR women OR female*)) ) AND ( exercis* OR (physical NEAR/0 activ*) OR (physical NEAR/0 train*) OR (aerobic NEAR/0 train*) OR (resistance NEAR/0 train*) OR (strength NEAR/0 train*) OR (aquatic NEAR/0 exercis*) OR (water-based NEAR/0 exercis*) OR pilates OR taekwondo OR (brain NEAR/0 gym) OR (circuit NEAR/0 train*) OR (multicomponent NEAR/0 exercis*) OR (multi-component NEAR/0 exercis*) OR (combined NEAR/0 train*) OR (mind-body NEAR/0 exercis*) ) AND ( cogniti* OR memory OR (executive NEAR/0 function*) OR MMSE OR "Mini-Mental State Examination" OR MoCA OR "Montreal Cognitive Assessment" OR "brain-derived neurotrophic factor" OR BDNF OR "body mass index" OR BMI ) AND ( random* OR trial* OR placebo* OR "controlled clinical trial" ) ) |
